# Headache and mechanical sensitization of human pericranial muscles after repeated intake of monosodium glutamate (MSG)

**DOI:** 10.1186/1129-2377-14-2

**Published:** 2013-01-24

**Authors:** Akiko Shimada, Brian E Cairns, Nynne Vad, Kathrine Ulriksen, Anne Marie Lynge Pedersen, Peter Svensson, Lene Baad-Hansen

**Affiliations:** 1 Section of Clinical Oral Physiology, Department of Dentistry, Faculty of Health Sciences, Aarhus University, Vennelyst Boulevard 9, Aarhus C 8000, Denmark; 2 Faculty of Pharmaceutical Sciences, University of British Columbia, 2405 Wesbrook Mall, Vancouver, BC V6T 1Z3, Canada; 3 Section of Oral Medicine, Clinical Oral Physiology, Oral Pathology & Anatomy, Department of Odontology, Faculty of Health Sciences, University of Copenhagen, Nørre Allé 20, Copenhagen, 2200 København N, Denmark; 4 MindLab, Center for Functionally Integrative Neuroscience, Aarhus University Hospital, Nørrebrogade 44, Aarhus C 8000, Denmark

**Keywords:** Monosodium glutamate, Craniofacial sensitivity, Pain, Accumulation, Headache

## Abstract

**Background:**

A single intake of monosodium glutamate (MSG) may cause headache and increased muscle sensitivity. We conducted a double-blinded, placebo-controlled, crossover study to examine the effect of repeated MSG intake on spontaneous pain, mechanical sensitivity of masticatory muscles, side effects, and blood pressure.

**Methods:**

Fourteen healthy subjects participated in 5 daily sessions for one week of MSG intake (150 mg/kg) or placebo (24 mg/kg NaCl) (randomized, double-blinded). Spontaneous pain, pressure pain thresholds and tolerance levels for the masseter and temporalis muscles, side effects, and blood pressure were evaluated before and 15, 30, and 50 min after MSG intake. Whole saliva samples were taken before and 30 min after MSG intake to assess glutamate concentrations.

**Results:**

Headache occurred in 8/14 subjects during MSG and 2/14 during placebo (P = 0.041). Salivary glutamate concentrations on Day 5 were elevated significantly (P < 0.05). Pressure pain thresholds in masseter muscle were reduced by MSG on Day 2 and 5 (P < 0.05). Blood pressure was significantly elevated after MSG (P < 0.040).

**Conclusion:**

In conclusion, MSG induced mechanical sensitization in masseter muscle and adverse effects such as headache and short-lasting blood pressure elevation for which tolerance did not develop over 5 days of MSG intake.

## Background

Temporomandibular Disorders (TMD) affect approximately 10% of the population [1,2]. The principal symptom of TMD that most often leads patients to seek medical treatment is pain in the temporomandibular joint and/or masticatory muscles [1]. Around 70% of TMD patients report masticatory muscle pain and are described as suffering from myofascial TMD [3]. In the majority of myofascial TMD cases, there is little evidence of ongoing pathological change in masticatory muscles, and, consequently, a number of alternative mechanisms have been proposed to explain pathogenesis of this pain [1,3]. Of these potential mechanisms, life-style related factors may play an important role in development and maintenance of muscle pain in myofascial TMD. For example, chronic stress has been speculated to lead to parafunctional activities such as repetitive tooth clenching or grinding that then produces strain injury to masticatory muscles leading to pain [4-6], although the relationship between bruxism and craniofacial pain may be more complex [7,8]. Diet may be another important life-style related factor contributing the muscle pain in myofascial TMD, as it has been reported that TMD patients alter their diet to avoid exacerbating pain associated with mastication of certain foods [9]. However, very little is currently known about the interaction between food intake and pain in TMD.

Consumption of certain foods is thought to precipitate or aggravate other chronic craniofacial pain conditions. One postulated food trigger for migraine headaches is monosodium glutamate (MSG), which is used as a taste enhancer in many snack and fast foods [10]. Indeed, total dietary consumption of glutamate ranges from 50–200 mg/kg/day [11,12]. Of note, healthy young men who consumed a single dose of 150 mg/kg MSG had a significant increase in headache and craniofacial muscle sensitivity as well as an elevated systolic blood pressure [13], suggesting that MSG consumption may trigger more types of craniofacial pain than just headaches. A significant amount of systemically administered glutamate is known to be taken up by skeletal muscles, including the masticatory muscles [14]. The resulting elevation of interstitial glutamate concentration sensitizes muscle nociceptors to mechanical stimuli [15]; an effect which could underlie reports of craniofacial muscle pain sensitivity in healthy young men given MSG [13]. Further, there is evidence that glutamate concentrations are elevated in painful regions of masticatory muscles of myofascial TMD patients compared to healthy controls [16].

Although a single 150 mg/kg dose of MSG did result in a demonstrable increase in craniofacial sensitivity in young healthy men, more regular consumption of this quantity of MSG may result in tolerance to the acute sensitizing effects of MSG [13]. On the other hand, chronic daily administration of 150 mg/kg of MSG might lead to the accumulation of MSG in masticatory muscles, which might be manifested by enhanced craniofacial sensitivity reminiscent of symptoms reported by myofascial TMD patients. The current study was therefore conducted to assess the impact of 5 days of consumption of 150 mg/kg MSG on craniofacial pain sensitivity in healthy subjects.

## Methods

### Study design and subjects

This study was performed as a double-blinded, placebo-controlled cross-over trial. The study protocol was approved by the local ethics committee (approval No. 20060040 – amendment No. 2 of March 2010) and informed consent was obtained from all subjects. The study was conducted in accordance with the Declaration of Helsinki. Randomization was performed by a computer and the examiners were blinded until after finishing data collection on all subjects.

Fourteen healthy adult (> 18 years) subjects (9 women and 5 men, mean age 27.6 ± 1.7 years, mean bodyweight 64.1 ± 2.5 kg) were included in the study through the webpage (http://www.forsoegsperson.dk) and among staffs and students at the Department of Dentistry, Aarhus University. Exclusion criteria were: orofacial pain, chronic illness, e.g. uncontrolled hypertension, allergy to MSG, asthma, diabetes mellitus, body mass index > 25 [13].

Each subject participated in 10 (2 × 5) sessions (Monday through Friday in two consecutive weeks). In one week, the subjects received MSG, and in the other week, placebo (see below), in randomized order.

### Baseline measurements

On the first day, the bodyweight of the subject was measured with the use of a digital scale. The subjects were asked to fast for at least 3 hours before each session. In each of the 10 sessions at baseline (BL), after a 10 min rest, a resting whole saliva sample was collected using the draining method [17]. The collection of saliva samples took place in a quiet room. The subjects were seated upright in a chair with the head slightly bent forward, and instructed to drool into a plastic cup for 10 min [18]. The subjects were then asked to rate spontaneous pain (if any) on a 0-10 numerical rating scale (NRS, 0 = no pain, 10 = most imaginable pain). Then, pressure pain thresholds (PPT) and pressure pain tolerance levels (PPTol) of the left masseter and temporalis muscles were measured [19]. Systolic and diastolic blood pressure (BP) and heart rate (HR) were also measured with the use of a digital blood pressure monitor (UA-767plus; A&D Medical, Abingdon, UK).

### Administration of monosodium glutamate (MSG) or placebo

In each session of the MSG week, a drink was prepared of 400 ml sugar-free lemon soda (Spirit light^®^, Coop) by a research assistant in a separate room. The beverage contained carbon dioxide, citric acid, sodium citrate, aromas, artificial sweeteners (aspartame, acesulfame potassium) and sodium benzoate. MSG (150 mg MSG per kg bodyweight) was added to the soda. In the placebo week, 24 mg per kg bodyweight of NaCl was added to the drink instead of MSG to create a similar taste. The drink was ingested over a few min [13].

### Pressure pain thresholds and pressure pain tolerance thresholds

An algometer (Somedic, Hörby, Sweden) was use to assess PPT and PPTol in the masseter and temporalis muscles. To undertake these measurements, the subject’s head was gently supported by the opposite hand of the examiner and the subject was instructed to keep his/her teeth slightly apart during the measurements [3,19]. Subjects were instructed to press a button when the force applied by the examiner just became painful (PPT) or when they could no longer tolerate the force being applied (PPTol). PPT (triplicates) and PPTol (single measures) for left masseter and left temporalis were repeated in all sessions 15, 30, and 50 min after drinking the beverage. Only the left side was assessed, since it has been shown that there are no overall differences between sides regarding pressure sensitivity [13].

### Self-reported pain and adverse effects

The subjects were asked to rate any pain on a 0-10 NRS at time-points 15, 30 and 50 min after the drink. In addition, they were asked about the following adverse effects: Headache, nausea, dizziness, feeling of chest pressure, burning sensation of skin, tiredness, sore jaw, stomach ache, and “other, please specify” [11,13]. They answered “yes” or “no” to each adverse reaction.

### Measurement of glutamate levels in resting whole saliva

Two resting saliva samples were collected in each session; one prior to and another 30 min after drinking the beverage. The whole saliva samples were pipetted into test tubes and placed on ice immediately after collection and then kept in a freezer (-80°C) until analysis. Each sample was centrifuged before measurements of glutamate (1500 g for 5 min). The concentration of glutamate in the whole saliva samples was determined with a commercially available kit (L-Glutaminsäure/L-Glutamate, Thermo Fisher Scientific Inc., Australia) using the photometric analyzer Konelab 30i (Thermo Clinical Labsystems/ILS Laboratories Scandinavia, Denmark). The molecular weight of glutamate is 146 g/mol. The standard (calibrator) in the kit was 0.1 g/L corresponding to 684.9 μM. Upper test limits for the kit was 3592.00 μM. A sample volume of 10 μL of saliva per measurement was used. Duplicate measurements were performed for each sample at a wavelength of 492 nm. Values of salivary glutamate were given in μM.

### Blood pressure and heart rate

BP and HR measurements with a digital blood pressure monitor (UA-767puls; A&D Medical, Abingdon, UK) were repeated in all sessions at 15, 30, and 50 min after drinking the beverage.

### Statistics

All data are presented as means ± standard error of the mean (SEM). Levels of *P* less than 0.05 were considered statistically significant. Spontaneous pain, PPT, PPTol, BP, HR, and glutamate concentration were analyzed with 3-way repeated measurement (RM) analyses of variance (ANOVAs) with intervention (MSG vs. placebo), day (Day 1-5 in each week) and time (BL, 15, 30, 50 min) as factors. For glutamate concentration only two time periods (BL and 30 min) were used. When appropriate, post hoc Tukey Honestly Significant Difference (HSD) tests with corrections for multiple comparisons were performed. Occurrence of side effects was compared between weeks with McNemar´s test.

## Results

### Spontaneous pain

**Figure 1:**
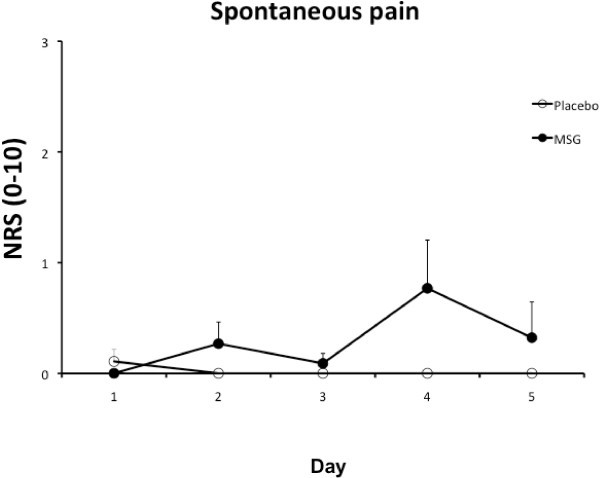
**The mean (± SEM) numerical rating scale (NRS) spontaneous pain reports from the subjects over 5 days of monosodium glutamate (MSG) and placebo intervention (N = 14).** Neither intervention resulted in significant spontaneous pain reports.

Three subjects experienced spontaneous pain in the craniofacial region in the MSG week (maximum pain level 6, 3 and 2, respectively) and one subject experienced pain (only on Day 1, maximum pain level 3) in the placebo week. However, group mean pain scores were low and not significantly influenced by intervention (MSG vs. Placebo), day (Day 1-5), or time (BL, 15, 30, 50 min after intake) (P > 0.174) (Figure 1).

### Pressure pain thresholds (PPT) and pressure pain tolerance (PPTol)

**Figure 2:**
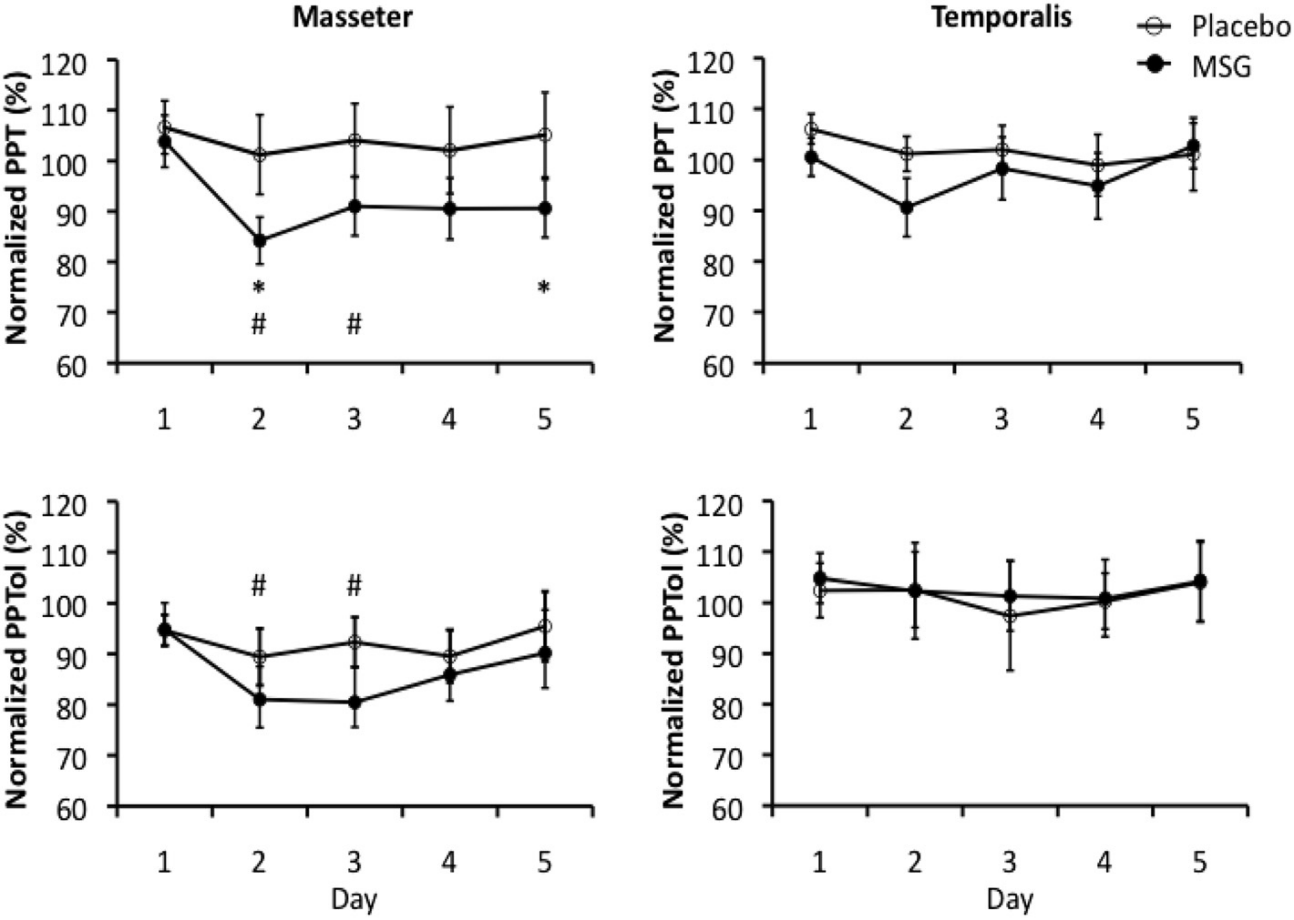
**The overall (± SEM) mean relative Pressure Pain Threshold (PPT) or Pressure Pain Tolerance (PPTol) for each day (N = 14).** There was a trend in PPT values in both muscles and PPTol values in the temporalis muscle to increase due to repeated measurement during each daily session, which resulted in mean overall values that exceeded 100% on all 5 days during placebo intervention. The masseter muscle PPT and PPTol measured on Day 2 and 3 were both significantly decreased compared to Day 1 (#: P < 0.05), but this difference occurred independent of intervention. The masseter muscle PPT was, however, significantly lower after monosodium glutamate (MSG) ingestion than after placebo ingestion on Day 2 and 5 (*: P < 0.05). There was no effect of intervention or day on the PPT and PPTol measured from the temporalis muscle.

PPT and PPTol measures were normalized to the BL value on Day 1 before analyses. There was no main effect of intervention (MSG vs. placebo) on PPT in left masseter (P = 0.159, F = 2.281). There was a main effect of day (P = 0.002, F = 4.996) and time (P = 0.007, F = 4.867) and a tendency towards a significant interaction between intervention and day (P = 0.079, F = 2.249). Post hoc tests revealed that left masseter PPT was significantly reduced on Day 2 and 3 compared to Day 1 (P < 0.048; Figure 2). Within sessions, left masseter PPT was increased at the 30 and 50 min time-points compared to BL (P < 0.032; Data not shown). Post hoc analysis of the interaction between intervention and day showed a significant reduction in normalized left masseter PPT on Day 2 and 5 in the MSG week compared to placebo and Day 1 (P < 0.029) (Figure 2). Left temporalis PPT was not influenced by intervention (P = 0.411, F = 0.729) or day (P = 0.337, F = 1.169) but there was a main effect of time (P = 0.002, F = 6.388) (Figure 2). Post hoc tests revealed that left temporalis PPT was increased at 15, 30 and 50 min compared with BL within sessions (P < 0.023; Data not shown).

There was no main effect of intervention (P = 0.645, F = 0.225) or time (P = 0.254, F = 1.420) on left masseter PPTol, but there was a main effect of day (P = 0.026, F = 3.054). The post hoc test showed a significant decrease in left masseter PPTol on Day 2 and 3 compared to Day 1 (P < 0.047) (Figure 2). Left temporalis PPTol was not significantly influenced by intervention (P = 0.943, F = 0.005) or day (P = 0.700, F = 0.550) but there was a main effect of time (P = 0.038, F = 3.156) (Figure 2). Left temporalis PPTol was significantly increased at 50 min compared with BL (P = 0.044; Data not shown).

### Adverse effects

**Figure 3:**
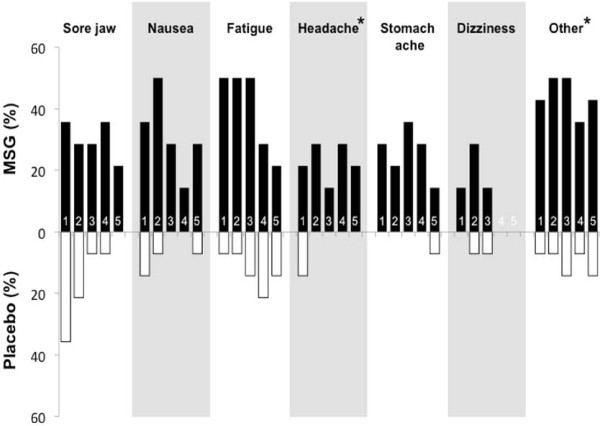
**The vertical bar chart shows the frequency of side effects reports by > 30% of the subjects over 5 days of monosodium glutamate (MSG) (black) and 5 days of placebo (white) intervention.** For most side effects, such as nausea and fatigue, there was a peak in reports on the second or third day of administration followed by a gradual decline in reports. There were substantially fewer reports of side effects in the placebo group, with the exception of reports of jaw soreness, which peaked during the first session and then declined. The Occurrence of headache and other side effects showed a significant difference between the interventions by McNemar’s test (*: P < 0.05).

A greater number of subjects reported adverse effects in the MSG week than in the placebo week (Table 1). The number of subjects who reported headache was significantly higher during the MSG week than during the placebo week. The daily incidence of the various side effects is shown in Figure 3. As can been seen in Figure 3, most side effects were more frequent at the beginning of intervention and tended to be less commonly reported in the final 2 days of MSG intervention. In contrast, some side effects, notably headache and stomach ache, had a similar frequency of occurrence throughout the entire 5 days MSG intervention.

**Table 1:** The percentage of subjects reporting side effects at least once over the 5 days of intervention

**Side effect**	**MSG**	**Placebo**	**McNemar’s test**
Nausea	57.1	21.4	P = 0.182
Headache*	**57.1**	**7.1**	**P = 0.041**
Dizziness	35.7	0.0	P = 0.074
Chest Pressure	28.6	0.0	P = 0.134
Burning	21.4	7.1	P = 0.617
Fatigue	50.0	28.6	P = 0.074
Sore Jaw	57.1	35.7	P = 0.450
Stomachache	35.7	7.1	P = 0.134
Other*	**57.1**	**14.3**	**P = 0.023**

Other included reports of numbness in appendages, feeling cold, and heartburn.

### Glutamate levels in whole saliva

**Figure 4:**
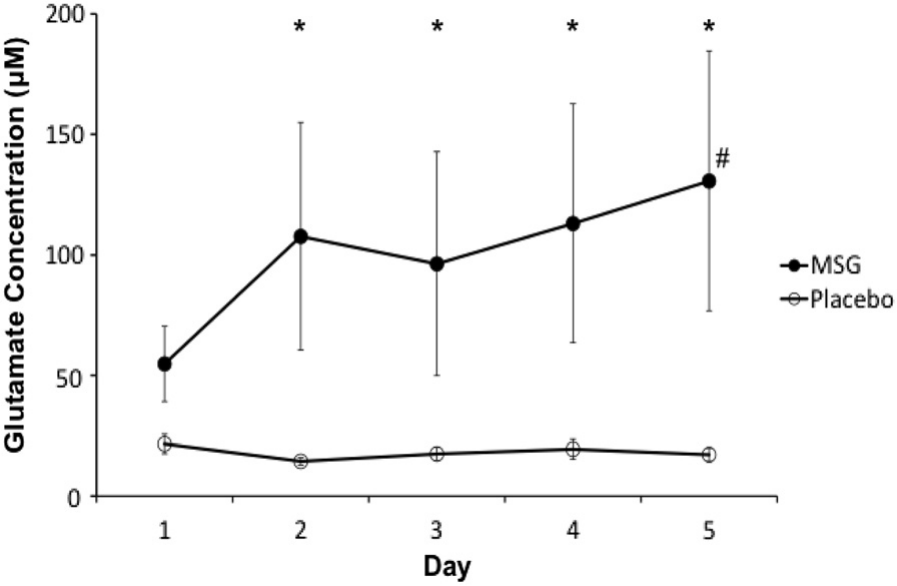
**The mean (± SEM) glutamate concentration in saliva during the monosodium glutamate (MSG) and placebo intervention (N = 14).** There was a significant increase in the concentration of glutamate in the saliva on Day 5, compared to Day 1 (#: P < 0.05). Note that there was a tendency for the glutamate concentration to increase each day of the 5 days MSG session, which suggests that a modest accumulation of glutamate was occurring. On Day 2 through Day 5, the concentration of glutamate was significantly higher in the MSG session compared to placebo session (*: P < 0.05).

Average trough (daily baseline) levels of salivary glutamate were 17 ± 3 μM in during the 5 days of MSG and 24 ± 4 μM during the 5 days of placebo. Average peak levels of salivary glutamate were 220 ± 61 μM in MSG week and 14 ± 2 μM in placebo week. The ANOVA of glutamate concentration demonstrated a significant interaction between intervention and day (P = 0.028, F = 3.010). Post hoc analyses showed that glutamate levels were significantly higher on Day 2 through Day 5 in the MSG week compared with the placebo week (P < 0.05). Also, in the MSG week, glutamate level on Day 5 was significantly higher than that on Day 1 (P < 0.05) (Figure 4).

### Blood pressure (systolic: sBP: diastolic: dBP) and heart rate (HR)

**Figure 5:**
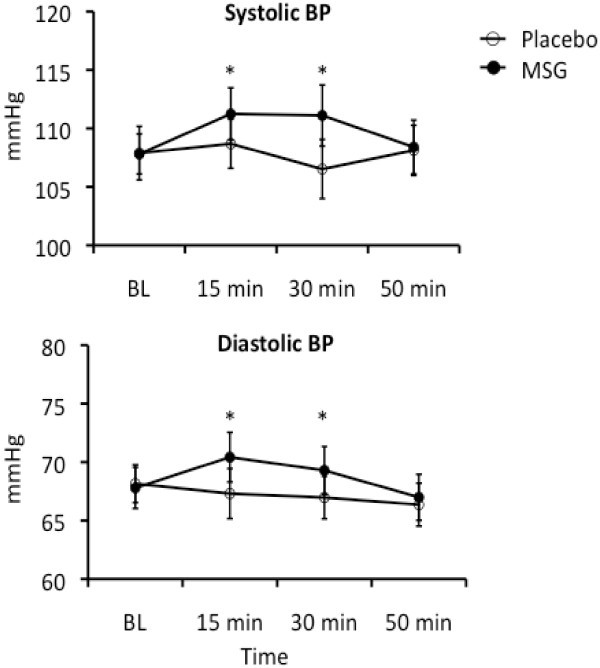
**The time course of changes in mean (± SEM) systolic and diastolic blood pressure during monosodium glutamate (MSG) and placebo intervention.** As there was no effect of day, a grand average of the results of all 5 days is shown. Note that there was a small, but significant (*: P < 0.05) increase in both systolic and diastolic blood pressure 15 and 30 minutes after ingestion of MSG, but no change in blood pressure after placebo.

There were no main effects of intervention (P = 0.079, F =3.727) or day (P = 0.324, F = 1.200) on sBP. However, there was a main effect of time (P = 0.026, F = 3.518) and a significant interaction between intervention and time (P = 0.007, F = 4.811). The post hoc test of the main time effect showed that sBP was elevated 15 min after intake (P = 0.022). A post hoc test of the intervention × time interaction revealed that in the MSG sessions (P < 0.040) but not in placebo sessions (P > 0.900), sBP was elevated 15 and 30 min after intake (Figure 5).

There were no main effects on dBP of intervention (P = 0.067, F = 4.132), day (P = 0.602, F = 0.692), or time (P = 0.099, F = 2.270). However, there was a significant interaction between intervention and time (P = 0.005, F = 5.049). The post hoc test indicated that dBP was significantly elevated in the MSG sessions at 15 and 30 min after intake compared with placebo sessions (P < 0.043) (Figure 5).

There were no main effects of intervention (P = 0.412, F = 0.728) or day (P = 0.265, F = 1.356) on HR, but there was a significant effect of time (P < 0.001, F = 10.104). HR decreased over time and was significantly lower than BL at 15, 30 and 50 min after intake of either substance (P < 0.003).

### Result of blinding procedure

At the end of each 5 days session, subjects were asked to indicate which substance they thought they had received. Subjects correctly identified the substance given to them 88% of the time, suggesting that due to the increased number of side effects experienced by many subjects who received MSG, most subjects were able to guess what they were receiving.

## Discussion

The present study was conducted to probe whether, compared to a single administration, repeated intake of MSG could lead to increased complaints of untoward affects and evidence of accumulation of glutamate. MSG (150 mg/kg) was administered daily for 5 days and pain sensitivity and side effects monitored. Daily intake of this amount of MSG did not result in significant reports of spontaneous pain. However, it did lead to a sustained mechanical sensitization of the masseter muscle that lasted for the duration of MSG administration. This pain sensitizing effect of MSG was not observed in the temporalis muscle, suggesting that the effect was site specific. Daily intake of MSG also caused headaches and dizziness for at least one day out of five in 57% of subjects. Although tolerance to most side effects of MSG administration appeared to occur, as evidenced by a decrease in the frequency of side effect complaints, the frequency of headache reports remained relatively constant over the 5 days of MSG ingestion. This suggests that tolerance to the headache inducing effects of MSG may not occur. There was also no apparent tolerance to the ability of MSG administration to increased systolic and diastolic blood pressure over the 5 days intervention. Baseline salivary concentrations of glutamate remained constant and were not different between the MSG and placebo sessions. These concentrations are similar to a previous study that reported mean saliva concentrations of glutamate of 18±1 μM in 18 subjects [20]. However, administration of MSG tended to increase salivary glutamate concentration each day, which suggests the potential that accumulation might have been occurring. These findings suggest that daily consumption of elevated amounts of MSG increases craniofacial pain symptoms in otherwise healthy subjects.

There are some differences in the response properties of the masseter and temporalis muscles to glutamate that may underlie the finding of a masseter muscle selective mechanical sensitizing effect of MSG. Elevated concentrations of glutamate induce mechanical sensitization of masticatory muscle nociceptors through activation of peripheral NMDA receptors [15,21]. It has been found that there is a lower expression of NMDA receptors by nociceptors that innervate the temporalis muscle and that the response of temporalis nociceptors to peripheral NMDA receptor activation is less robust than masseter muscle nociceptors [22,23]. Thus, it is possible that there is also a lower expression of peripheral NMDA receptors by nociceptors that innervate the temporalis muscle in healthy human subjects, which results in the temporalis muscle being less sensitive to orally consumed MSG than the masseter muscle [24].

There was a very consistent, albeit relatively small, increase in both systolic and diastolic blood pressure after oral intake of MSG each day. There is recent evidence that increased dietary intake of glutamate over a 5 year period is correlated with an increase in systolic blood pressure, which was more pronounced in women than in men [25]. It is also important to consider whether MSG-induced increases in blood pressure account for the increased incidence of headaches observed in the present as well as previous studies [13,14]. Although earlier work suggested that elevated blood pressure causes headaches [26-28], more recent work does not support a strong association. For example, in a large population of Icelandic men and women, it was found that elevated systolic blood pressure was inversely correlated with migraine headache in both men and women [29]. There is also no difference in mean or systolic blood pressure in women with chronic daily headache and women without this condition [30]. These findings suggest that mechanisms other than hypertension may explain MSG-induced headaches.

Glutamate has also been shown to dilate intracranial and extracranial blood vessels through a peripheral NMDA receptor mechanism that involves the release of nitric oxide [31-33]. Many therapeutically employed vasodilators appear to cause headaches as one of their side effects [34]. For example, infusion of nitroglycerin reliably induces a headache, which is thought to be due to dilation of extracerebral arteries [35,36]. In a recent study in migraine headache patients, infusion of calcitonin gene related peptide (CGRP), a potent neuropeptide vasodilator and migraine headache inducer, was shown to induce vasodilation of arteries only on the side of the migraine headache pain [37]. On the other hand, vasodilation itself is not able to induce pain that occurs only when periovascular nerve endings are sensitized. This shows that a neural factor, i. e. sensitization, seems to be related to chronic MSG administration [38]. We speculate that it could be vasodilation of extracranial blood vessels through peripheral NMDA receptor activation that mediates MSG headaches [39].

Baseline salivary concentrations of glutamate did not increase over the 5 days of MSG intake, however, post MSG glutamate concentrations increased over the 5 day period. Glutamate has an apparent half-life of about 30 min, which suggests that it would be completely cleared from the blood within 4 hrs of ingestion of MSG [14]. Previously, it was reported that a single oral dose of 150 mg/kg MSG resulted in peak blood glutamate concentrations of a little over 400 μM, which occurred 30-45 min after ingestion [14]. This suggests that salivary glutamate concentrations are about 25-50% of blood concentrations. It has been suggested that as much as 40% of a 150 mg/kg oral dose of MSG is removed by and stored in skeletal muscle [14,40]. Increased peak levels in the saliva over the 5 days could reflect saturation of storage sites in the skeletal muscle. Animal research suggests that after systemic administration of MSG (50 mg/kg i.v.) there is a rapid rise in glutamate concentration in the masseter muscle that is associated with significant mechanical sensitization of muscle nociceptors [15]. It is likely that oral MSG consumption increases glutamate concentration in the masseter muscle of human subjects and that this underlies the mechanical sensitization of the masseter muscle seen in the present study.

Although the MSG was dissolved in sugar free lemon soda, which we have previously found masks the taste of MSG [13], the vast majority of subjects correctly identified the substance administered to them when asked at the end of each 5 days session. Considering the significant increase in adverse effects, which occurred during MSG ingestion, this result is understandable. Nevertheless, it does mean that we cannot consider this study truly blind, and this lack of subject blinding might have influenced findings that relied on psychophysical assessments, such as PPT and PPTol, and reporting of side effects. However, it should be noted that even though PPT and PPTol were assessed in two jaw closing muscles, significant differences were only found for the masseter muscle. This suggests that even if subjects thought they knew what they were receiving their responses were not reflective of any systematic bias. Also, the administration of placebo can induce adverse effects such as headache, which complicates clarification of adverse reactions induced by MSG [41]. Though adverse effects of headache in the placebo week also could be observed in the result of the present study, it was seen to a significantly lesser extent than in the MSG week. This may imply that an accumulation of MSG by oral administration could be a factor best avoided by TMD and headache patients. Mechanical sensitization of masseter muscles is one of the typical symptoms of TMD and there is a well-known overlap between painful TMD and headache [42].

## Conclusion

In conclusion, the present study suggests that individuals who consume elevated amounts of MSG in their diet are more likely to suffer from headaches and have masseter muscle sensitivity, two symptoms associated with myofascial TMD. Although far from definitive, the present findings may indicate that there is a link between diet and susceptibility to the development of chronic orofacial pain conditions that include TMD and headache. If future studies can confirm a link, dietary modification could become an important part of treatment aimed at ameliorating symptoms associated with these chronic craniofacial pain conditions.

## Competing interest

The authors declare that there are no conflicts of interest in the publication of this manuscript.

## Authors’ contributions

AS participated in collecting data and drafted the manuscript. BEC conceived of the study, and participated in its design and drafted the manuscript. NV and KU took a part in collecting data and performed the statistical analysis. AMLP carried out the analysis of the saliva samples. PS supervised this study, participated in its design and coordination and helped to draft the manuscript. LBH participated in the design of the study, planned the data collection, performed the statistical analysis, and drafted the manuscript. All authors read and approved the final manuscript.
